# *Alphaherpesvirus* glycoprotein E: A review of its interactions with other proteins of the virus and its application in vaccinology

**DOI:** 10.3389/fmicb.2022.970545

**Published:** 2022-08-04

**Authors:** Yaru Ning, Yalin Huang, Mingshu Wang, Anchun Cheng, Qiao Yang, Ying Wu, Bin Tian, Xumin Ou, Juan Huang, Sai Mao, Di Sun, Xinxin Zhao, Shaqiu Zhang, Qun Gao, Shun Chen, Mafeng Liu, Dekang Zhu, Renyong Jia

**Affiliations:** ^1^Research Center of Avian Disease, College of Veterinary Medicine, Sichuan Agricultural University, Chengdu, Sichuan, China; ^2^Institute of Preventive Veterinary Medicine, Sichuan Agricultural University, Chengdu, Sichuan, China; ^3^Key Laboratory of Animal Disease and Human Health of Sichuan Province, Chengdu, Sichuan, China

**Keywords:** *alphaherpesvirus*, glycoprotein E, interaction, viral protein, vaccination

## Abstract

The viral envelope glycoprotein E (gE) is required for cell-to-cell transmission, anterograde and retrograde neurotransmission, and immune evasion of *alphaherpesviruses*. gE can also interact with other proteins of the virus and perform various functions in the virus life cycle. In addition, the *gE* gene is often the target gene for the construction of gene-deleted attenuated marker vaccines. In recent years, new progress has been made in the research and vaccine application of gE with other proteins of the virus. This article reviews the structure of gE, the relationship between gE and other proteins of the virus, and the application of gE in vaccinology, which provides useful information for further research on gE.

## Introduction

The herpesvirus family is subdivided into the *alphaherpesvirinae*, *betaherpesvirinae* and *gammaherpesvirinae*. Of these, *alphaherpesviruses* have the broadest host range and can establish latent lifelong infections in sensory neurons (Hernández Durán et al., 2019). Reactivation from a latent state produces new infectious virus particles that travel along axons or dendrites to peripheral tissues (epithelial tissue) and higher-level neurons. The *alphaherpesvirus* subfamily includes herpes simplex virus type 1 (HSV-1), herpes simplex virus type 2 (HSV-2), pseudorabies virus (PRV), varicella zoster virus (VZV), bovine herpesvirus 1 (BHV-1), equine herpesvirus 1 (EHV-1), Marek’s disease virus (MDV) and duck enteritis virus (DEV; Yuan et al., 2005; Chang et al., 2009; Wu et al., 2012a,b; Vallbracht et al., 2019; Xu et al., 2020; Liao et al., 2021).

*Alphaherpesviruses* have a common and unique virion morphology (Davison et al., 2009). The linear double-stranded DNA genome is wrapped in an icosahedral capsid consisting of 150 hexagons and 12 pentagons, forming the nucleocapsid. The nucleocapsid is embedded in a protein layer called the tegument, which in turn is surrounded by a host cell-derived lipid bilayer, the envelope. The viral envelope contains multiple proteins, mainly glycoproteins, of varying copy numbers (Davison et al., 2009; Mettenleiter et al., 2009). Twelve glycoproteins of *alphaherpesviruses* have been identified, including gB, gC, gD, gE, gH, gI, gG, gK, gL, gM, gN and gJ (Chang et al., 2010, 2011a,b; Lian et al., 2010, 2011; Zou et al., 2010; Zhang et al., 2010b,c, 2011; Jambunathan et al., 2011; Li et al., 2011; Wang et al., 2012; Lin et al., 2013, 2014; Sun et al., 2013, 2014; You et al., 2018; Zhao et al., 2019). Different glycoproteins have different functions, recognizing and interacting with specific receptors on the cell surface, mediating fusion of the viral lipid envelope with the cell membrane, completing secondary vesicles at trans-Golgi network (TGN)-derived vesicles membrane coating produces mature virions, which are subsequently released to the outside by membrane fusion (Browne et al., 2004; Campadelli-Fiume et al., 2012; Oliver et al., 2013, 2017; Heldwein, 2016; Rider et al., 2017; Vallbracht et al., 2018).

The glycoprotein gE is encoded by the *US8* gene, which is the major virulence determinant of the virus and has been tested in vaccine development strategies against the virus. The gE is not essential for viral replication, but it can interact with other proteins to facilitate the secondary envelope coating of virions, cell-to-cell transmission, and enhance the neurovirulence of the virus. Importantly, gE normally forms a heterodimer with the gI encoded by the *US7* gene, and the gE/gl heterodimer can participate in multiple functions of the virus. The gE/gI heterodimer can also interact with the Fc fragment of immunoglobulin G (IgG) to regulate the phosphorylation of extracellular regulated protein kinases 1/2 (ERK1/2), which facilitates immune evasion by the virus after infection. In addition, gE is the preferred target gene for the construction of attenuated vaccines. In recent years, some changes in the *gE* gene have promoted the enhancement of PRV virulence, and the *gE* gene is considered to be the main target for PRV vaccine development (Dong et al., 2018). This article summarizes the structure of the glycoprotein gE, the interaction between gE and other proteins of *alphaherpesviruses*, and the latest progress of gE-deleted marker vaccines, which provides a reference for the research of *gE* gene.

## gE structure and gE/gI heterodimer

gE is the main component of the viral envelope, and the function of gE largely depends on its structure. gE is a type I membrane protein characterized by a single passage of a polypeptide chain across the membrane. According to its transmembrane characteristics, gE is divided into three parts: extracellular domain (ETD), transmembrane domain (TMD) and cytoplasmic domain (CTD).

The CTD of gE includes a phosphorylated acidic amino acid cluster associated with Golgi localization (Zhu et al., 1996), a casein kinase II phosphorylation sequence consisting of two serine and two threonine residues (or sites; Olson et al., 1997), a tyrosine endocytosis motif YXXØ (Y is tyrosine, X is any amino acid, and Ø represents any large hydrophobic amino acid residue; Tirabassi and Enquist, 1999). These motifs are involved in the secondary envelope of the virus and distribute newly produced virions to cell junctions, allowing the virus to spread from cell to cell. Tyrosine-based targeting motifs and casein kinase II phosphorylation sequences within different glycoproteins display similar functions. The tyrosine-based targeting motifs YTQV, TS and YXXL in the HSV-1 gB CTD, TS in the gE CTD play similar functions in intracellular sorting and endocytosis. HSV-1 UL13 binds to gE and mediates the phosphorylation of gE through the casein kinase II phosphorylation sequence (Ng et al., 1998; Ortiz et al., 2004; Beitia Ortiz de Zarate et al., 2007; Koyanagi et al., 2018).

The conserved gE ETD is critical for the formation of the gE/gI heterodimer, which plays an important role in cell-to-cell spreading and immune evasion. Heterodimeric gE/gI accumulates early in the Golgi apparatus and distributes to cellular junctions later in viral infection and then to the extracellular side (Farnsworth and Johnson, 2006). This process occurs mainly between polarized cells, such as epithelial cells and neuronal cells (Mo et al., 2000; Collins and Johnson, 2003; Polcicova et al., 2005). Binding of gE ETD to IgG upon virus infection of cells results in redistribution of viral surface glycoproteins, thereby facilitating virus infection of adjacent uninfected cells by a gE-dependent mechanism (Rizvi and Raghavan, 2003). The interaction domain between gI and gE of HSV-2 (129 ~ 145 aa), and the interaction domain between gE and gI (231 ~ 260 aa) is more conserved than other domains, indicating that the formation of gE/gI heterodimer is an important event (Rowe et al., 2019). The ETD of PRV gE (1 ~ 122 aa) and the ETD of gI (1 ~ 106 aa) can form a gE/gI complex (Tyborowska et al., 2006). Furthermore, conserved cysteine-rich regions (C1 and C2) in the gE ETD are critical for the formation of the gE/gI heterodimer. The C2 region of BHV-1 gE is not necessary for the formation of the gE/gI complex. When gE was further truncated and the C1 region was deleted, it could not form a complex with gI, indicating that the C1 region of gE was important for the gE/gI complex formation is required (Tyborowska et al., 2000). Deletion of the first cysteine-rich region (208 ~ 236 aa) in the VZV gE ETD abolished gE/gI complex formation, which in turn affected gE/gI distribution on the plasma membrane and cell-to-cell diffusion of infected cells (Berarducci et al., 2009). 105 ~ 125 aa of VZV gI, aa at position 95, is critical for gE/gI heterodimer formation, virion incorporation, and virulence (Oliver et al., 2011). The above studies demonstrate that the conserved cysteine residues in the gE/gI ETD are critical for gE/gI heterodimer formation, virion incorporation and efficient virus transmission.

**Figure 1:**
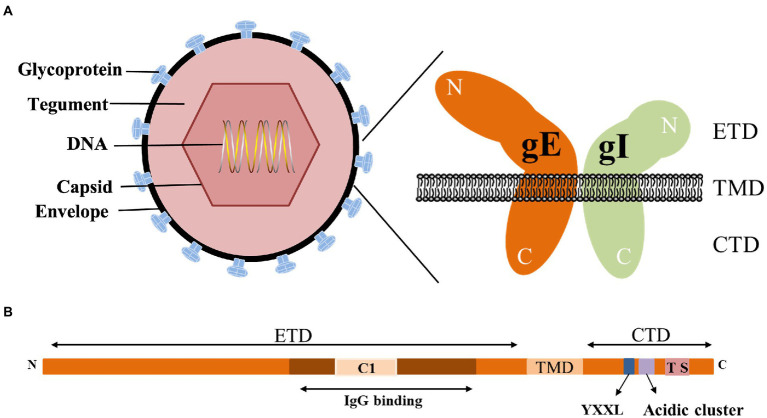
Structure of the *alphaherpesvirus* gE. **(A)** Structure of *alphaherpesviruses* and gE/gI heterodimer (DuRaine et al., 2017). **(B)** Main features of gE identified in this paper (Polcicova et al., 2005; Farnsworth and Johnson, 2006; Sprague et al., 2006).

The gE/gI heterodimer can interact with the Fc fragment of IgG. By analyzing the crystal structures of gE and gE/gI-Fc complexes, it was shown that the C-terminal domain of gE ETD is the smallest Fc-binding domain, and gE binds Fc at the CH2-CH3 interface (Sprague et al., 2006). The occupation of the IgG Fc region by the gE/gI complex inhibits the classical complement activation pathway and protects virus-infected cells from antibody-dependent cell-mediated cytotoxicity and antibody-dependent cellular phagocytosis (Lubinski et al., 2011; Bournazos et al., 2015; Sun et al., 2017; Jenks et al., 2019; Figure 1).

## Interaction of gE with tegument proteins

**Figure 2:**
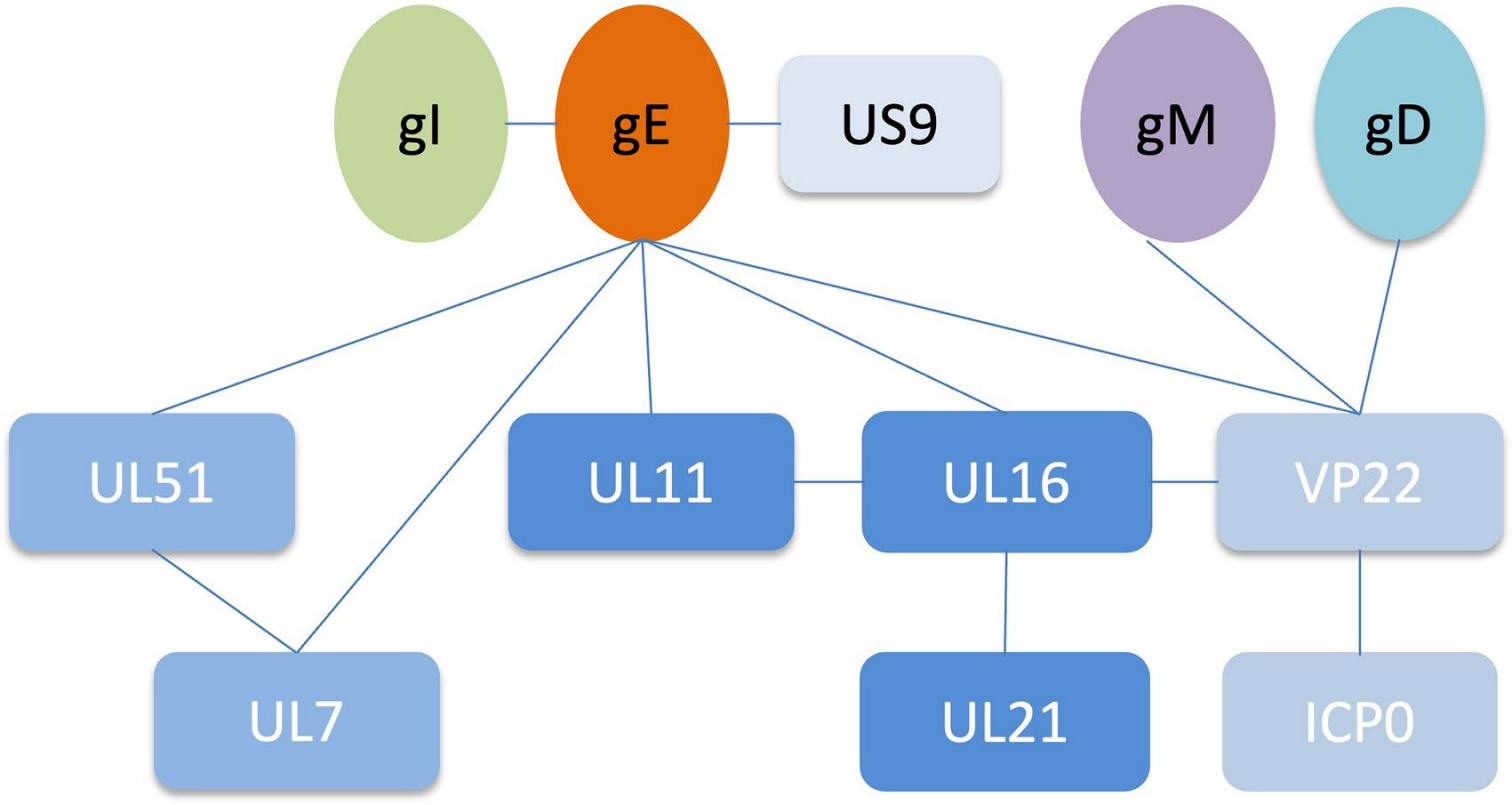
Network of protein–protein interactions around the HSV-1 glycoprotein E (Han et al., 2012; Maringer et al., 2012; Owen et al., 2015; Feutz et al., 2019).

*Alphaherpesviruses* encode 23 envelope proteins that play diverse roles in primary infection, secondary envelope, cell-to-cell spread, and immune evasion (Oda et al., 2016; Dai and Zhou, 2018; Hernández Durán et al., 2019). The encoded tegument proteins of HSV-1 range in size and abundance, with the smallest predicted to be approximately 10.5 kDa (UL11) and the largest greater than 335 kDa (UL36). It has been reported that some tegument proteins interact with glycoproteins to assist in the secondary envelope coating of virions, facilitate viral transport to cell junctions and indirectly aid in the spread of virions between cells, including gH and VP16, gK and UL37, gD with UL16 (Kamen et al., 2005; Jambunathan et al., 2014; Carmichael et al., 2019; Yang et al., 2021a,b). Deletion of gE resulted in massive capsid accumulation around the vesicles, severely inhibiting virion formation, suggesting that gE plays an important role in the secondary envelope coating of the cytoplasmic nucleocapsid (Liu et al., 2020). Interactions between gE and tegument proteins mediate multiple functions during the viral life cycle. gE interacts with other tegument proteins to form complexes that promote the secondary envelope, which facilitates virus-infected cell-to-cell fusion and releases mature virions (Table 1; Figure 2). In addition to the proteins listed in Table 1, the results of bioinformatics, immunoaffinity purification and mass spectrometry further indicated that the proteins gE, US10 and UL37 were involved in the recruitment of capsids to cytoplasmic vesicles, as well as ICP0, ICP4 and UL46 recruitment to form virions (Hernández Durán et al., 2019).

**Table 1:** Properties and functions of gE-interacting envelope proteins in HSV-1.

HSV-1	Interaction partners	Function	References
gE	UL11-UL16-UL21	UL11 promotes the interaction between gE and UL16. gE accumulates at the plasma membrane in the presence of UL11, UL16 and UL21. Role in cell-to-cell spread and cell fusion.	Chadha et al., 2012; Han et al., 2012; Carmichael et al., 2019
	UL7-UL51	Role in the distribution of gE at junctional surfaces of cells, cell-to-cell spread and cell fusion.	Roller et al., 2014; Feutz et al., 2019
	VP22	Affect the localization and packaging of the virus in the Golgi apparatus. Comprised a multicomponent complex with gE, gM, gI and the tegument proteins VP22, ICP0 to promote cell-to-cell spread and secondary envelopment.	Stylianou et al., 2009; O'Regan et al., 2010; Maringer et al., 2012; Starkey et al., 2014

### Interaction of gE with UL11-UL16-UL21

UL11 is a myristylated tegument protein with multiple sequences including myristate and nearby palmitate moieties, a leucine-isoleucine motif, an acidic cluster. UL11 is localized to various membrane structures of infected cells, such as the cell membrane and Golgi apparatus. UL11 binds to the cytoplasmic side of the host membrane via an N-terminal myristate and a nearby palmitate moiety. UL11 is required for efficient membrane fusion events during virus entry and spread (Kim et al., 2013). The amino acid sequence of UL16 protein is conserved, and it contains a nuclear localization signal motif at the N-terminus of the protein. The role of UL16 is to facilitate viral replication, nuclear egress of the capsid and final envelopment of the capsid on the cytoplasmic membrane. Deletion of UL16 in HSV-2 results in an approximately 50 ~ 100 times replication defect of the virus, accompanied by a defect in the export of the capsid nucleus and a secondary envelope defect in the cytoplasmic capsid. Analysis of the virion composition after UL16 deletion revealed a decrease in the packaging of gE (Starkey et al., 2014; Gao et al., 2018). UL21 is a conserved protein with many important functions in RNA binding, viral replication, cytoplasmic capsid budding, trafficking, syncytia formation and cell-to-cell transmission, but its function varies slightly in different viruses (Metrick et al., 2015; Metrick and Heldwein, 2016; Sarfo et al., 2017). Compared with HSV-1, the UL21 mutant had a greater effect on the cell-to-cell spread of HSV-2 (Finnen and Banfield, 2018). HSV-2 UL21 mutation results in delayed expression of immediate early viral genes and the formation of large DNA-containing capsids in the nucleus (Le Sage et al., 2013). Moreover, the decreased ability of DNA-containing capsids to exit the nucleus of infected cells caused by HSV-2 UL21 mutation was similar to that of UL16 mutation, suggesting that UL16 and UL21 proteins may work together to promote the nuclear export of capsids (Gao et al., 2017). The gE CTD interacts with the acidic cluster of UL11, which is required to activate the gE-UL16 interaction. UL16 interacts with UL21, which changes the conformation of UL16, thereby activating the UL11-UL16 interaction (Chadha et al., 2012; Han et al., 2012; Yang et al., 2020, 2021a). UL11 and UL21 do not interact directly, but can interact indirectly through UL16. gE and the envelope proteins UL11, UL16, UL21 form complexes that promote secondary envelopes that aid in cell fusion and thus facilitate viral spread from infected cells to adjacent uninfected cells (Owen et al., 2015; Carmichael and Wills, 2019). The capsid has the TGN as the primary secondary envelope site, and the capsid is usually found in close proximity to the viral glycoprotein at the TGN. UL11, UL16 and UL21 are transported to the Golgi through the localization motif of UL11, and gE, UL11, UL16, UL21 form a quadruplex to facilitate the secondary envelope of the Golgi. UL11 then determines the accumulation of the gE-UL11-UL16-UL21 complex on the plasma membrane of infected cells, thereby promoting cell fusion, cell-to-cell spreading, and release of mature virions.

### Interaction of gE with UL7-UL51

*Alphaherpesvirus* proteins UL7 and UL51 are envelope components that play a role in viral assembly and cell-to-cell transmission. Changes in viral titer and plaque diameter, and electron microscopy of UL7 deficient PRV infected cells revealed that UL7 is associated with secondary envelope and release of mature virions (Fuchs et al., 2005). UL51 and UL11 have similar structure and function. UL51 contains a membrane localization motif associated with Golgi localization and a conserved YXXL motif, which provides lipid anchors and facilitates protein binding to cell membranes. Mutation of the conserved YXXL motif in the cytoplasmic tail of UL51 disrupts the formation of viral assembly compartments in the neuronal cytoplasm, and UL51 affects the accumulation of conserved proteins located at cell surface junctions, affecting viral assembly and cell-to-cell transmission (White et al., 2020). UL51 is palmitoylated at the N-terminal cysteine, thereby localizing UL51 to the Golgi apparatus (Nozawa et al., 2003). Mutation of the phosphorylation site in UL51 significantly reduced viral replication, disrupted the membrane adhesion state and affected the cell-to-cell spread of the virus (Kato et al., 2018). HSV-1 UL51 forms a complex with UL7 in infected cells and, in the absence of other viral proteins, affects the accumulation of UL7 on the plasma membrane and thus the ability of the virus to spread from cell to cell (Roller and Fetters, 2015). Cells aggregated more easily in the absence of HSV-1 UL7-UL51, suggesting that the UL7-UL51 complex is important for maintaining cell morphology and regulating focal adhesion activity (Albecka et al., 2017).

HSV-1 gE-UL7-UL51 forms functional complexes and localizes at cell surface junctions where gE can accumulate and form syncytia. Deletion of amino acids 167 ~ 244 of UL51 results in the inability of gE to concentrate on the junction surface of Vero cells. By analyzing the transmission phenotype of the gE and UL51 double deletion viruses, it was found that gE and UL51 have the same transmission pathway in HaCaT cells (Feutz et al., 2019). Compared with gE deletion, HSV-1 UL51-depleted virus was more defective in cell-to-cell spread, suggesting that UL51 has a gE-independent function in epithelial cell spread (Roller et al., 2014). The above proves that complexes such as viral glycoprotein gE/gI and involucrin UL7-UL51 are concentrated on the cell surface, affecting the morphology of virus-infected cells, stabilizing the focal adhesions of cell membranes, and providing physical structural support for cells.

### Interaction of gE with VP22

VP22 is encoded by the *UL49* gene and is one of the most abundant involucrin proteins in herpesviruses. VP22 regulates the translocation of multiple viral and cellular proteins, promoting neurovirulence, viral spread, and cell cycle regulation (Tanaka et al., 2012; Trapp-Fragnet et al., 2019). HSV-1 VP22 interacts with cGAS, reduces cGAS activity and promotes VP22 replication, thereby continuing to evade the host’s innate antiviral response (Huang et al., 2018).

gE interacts with the tegument protein VP22 and affects viral localization and packaging in the Golgi apparatus. Yeast two-hybrid assays demonstrated that the CTDs of gE and gM interact specifically with the C-terminus of PRV VP22 (Fuchs et al., 2002). There are 14 amino acids in VP22 that are key regions for binding to gE. After deletion, VP22 cannot bind to gE, resulting in the inability of VP22 to be recruited to the cytoplasmic transport complex. The growth phenotype is identical to that produced by deletion of the entire VP22 gene sequence (Stylianou et al., 2009). Clusters of acidic amino acids in HSV-1 VP22 can interact with components of the clathrin sorting machinery of the Golgi apparatus to facilitate virion incorporation (O'Regan et al., 2010). VP22 interacts with gM, UL49.5 and UL16 to facilitate viral cell-to-cell spread and virion incorporation (Starkey et al., 2014; Pannhorst et al., 2018). The glycoproteins gE, gM, gI and the tegument protein VP22, ICP0 can form a multicomponent complex, and the double deletion of gE and gM greatly reduces the number of VP22 and ICP0 involved in virion assembly, resulting in smaller plaque size (Maringer et al., 2012).

## Interaction of gE/gI with envelope protein US9

The envelope protein US9 is involved in anterograde axonal transport of viruses in neurons, axonal sorting and assembly of mature virions (Lyman et al., 2007; Taylor et al., 2012; Daniel et al., 2015; Miranda-Saksena et al., 2015). US9 is a type II membrane protein containing a conserved domain including an arginine residue, an acidic domain and two conserved serine phosphorylation sites. Arginine residues within the conserved domain of HSV-1 US9 are critical for binding the molecular motor kinesin-1, which contributes to anterograde axonal transport and spread of viral particles from neurons to skin (Diefenbach et al., 2016). Axonal anterograde transport requires the acidic domain of BHV-1 US9. When BHV-1 enters the body through the nose and eyes, it establishes a latent lifelong infection in the trigeminal ganglion (TG). Mutant BHV-1 lacking the acidic domain of US9 (residues 83 to 90) was not detected in nasal and ocular swabs of animals when the virus was reactivated from latency (Chowdhury et al., 2011). Two conserved serine phosphorylation sites are required for anterograde diffusion, and most phosphorylated US9 proteins aggregate into axonal vesicles and lipid raft membranes. Two conserved serine phosphorylation sites are required for anterograde diffusion, and phosphorylated US9 protein accumulates in axonal vesicles and lipid raft membranes, enhances US9-Kif1-A binding, and enhances virion in axons efficiency of anterograde propagation over medium and long distances (Kratchmarov et al., 2013b). The association of US9 with cell surface lipid rafts is critical for the directional spread of PRV from presynaptic to postsynaptic neurons and for anterograde spread of infection (Lyman et al., 2008). Anterograde axonal transport of capsids (not glycoproteins) to distal axons is reduced, and despite the presence of key envelope glycoproteins, there is a secondary defect in viral assembly in distal axons in the absence of pUS9 (Miranda-Saksena et al., 2015).

gE/gI plays a role in anterograde axonal transport of virions to distal axons and extracellular virions from neurons to adjacent epithelial cells. In the absence of PRV gE/gI, viral particles assemble within the cell body and cannot be efficiently sorted into axons (Kratchmarov et al., 2013a). The CTD of gE facilitates the physical connection between gE/gI and microtubule motors in the Golgi apparatus and facilitates the loading of progeny virions or virion components on microtubules for axonal transport. BHV-1 gE CTD truncated virus was not efficiently retrogradely transported from the TG to the nose and eyes (Liu et al., 2008; Chowdhury et al., 2010). The gE ETD plays a key role in both anterograde axonal transport of virions to distal axons and the spread of extracellular virions from neurons to adjacent epithelial cells. The two mutant strains, gE-277 and gE-348, had different insertions in the gE ETD, neither of which affected anterograde axonal transport of virions, but gE-277 was unable to transmit virions from neurons to epithelial cells (Howard et al., 2014).

**Figure 3:**
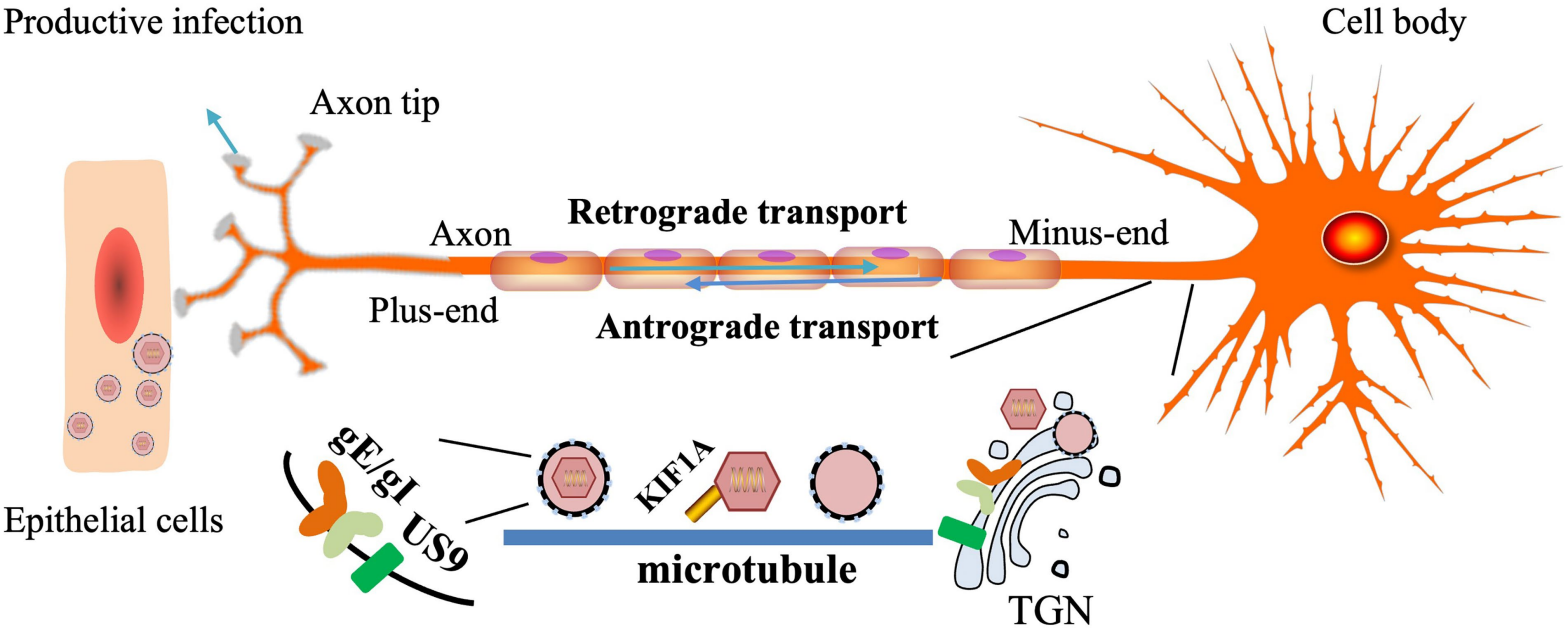
Models for how HSV gE/gl and US9 promote axonal transport (Howard et al., 2013, 2014). The TGN membrane vesicles containing gE/gI and US9 present in neuronal-cell bodies are adjacent to microtubules and serve as platforms for the assembly or loading of tegument-coated capsids onto microtubule motors.

gE/gI binds to US9 and forms a trimolecular complex in virus-infected cells, but the interaction between US9 and gE is easily disrupted by ionic detergents (Awasthi and Friedman, 2016). Intact axonal, gE, gI, and US9 proteins are required for virus transmission in neurons to cells, and capsids wrapped in vesicles can be found along the entire length of axons during viral infection (Ch'ng and Enquist, 2005). HSV gE/gI and US9 promote capsid and other glycoprotein trafficking in axons, respectively, and load capsid and glycoprotein-containing vesicles onto microtubule motors, thereby delivering HSV structural components to axon tips (Snyder et al., 2008). HSV mutants lacking gE and US9 are unable to properly assemble enveloped virions in the cytoplasm, sort virions, and transport virions to proximal axons (Howard et al., 2013; DuRaine et al., 2017). In addition, gE-gI-US9 formed a tripartite complex to recruit KIF1A, and US9 could accelerate the rate of KIF1A (Scherer et al., 2020). gE/gI is required for efficient anterograde transport of viral particles in axons by mediating the interaction between US9 and KIF1A early in infection, and when the interaction between US9 and KIF1A is weakened, the capsid stalls in in the axon (Kramer et al., 2012). The US9-gE/gI protein complex recruits KIF1A to viral transport vesicles for axonal sorting and transport, while promoting proteasomal degradation of the intrinsically labile KIF1A protein in axons, resulting in progeny particles in the time for transmission between host cells is limited (Huang et al., 2020). The study found that PRV did not spread anterogradely in the absence of gE or gI (but not US9), suggesting that PRV gE and gI proteins are essential for anterograde spread (McGraw et al., 2009; Figure 3).

## Deleted marker vaccines designed with gE

As mentioned above, gE interacts with a variety of proteins and participates in a variety of activities. gE participates in the secondary envelope and transports virions to the surface of infected cells for cell-to-cell spread, thereby facilitating virion maturation and spread between susceptible cells. The gE promotes virus to transmit from primary neurons such as the retina, TG to central nervous tissues, and plays a decisive role in axonal transport and neuroinvasiveness. Furthermore, gE/gI is closely related to immune evasion. Through sequence alignment, it was found that the amino acids of some proteins (such as gB, gC, and gE proteins) in the Chinese PRV strains have changed, resulting in the inability of existing vaccines to provide adequate protection (Wang et al., 2015b; Sun et al., 2018). Further research found that two recombinant viruses (rZJ01-LA/gEI and rLA-ZJ01/gEI) were successfully constructed by exchanging the *gE* and *gI* genes of the PRV mutant ZJ01 with the parental strain LA. rLA-ZJ01/gEI exhibited higher virulence than its parental virus, rLA (Dong et al., 2018). The *gE* gene is important for the virulence of the virus and is not necessary for the replication of the virus, and the alteration of the gE gene is part of the reason for the increase in the virulence of the virus in recent years. At present, the main means of virus prevention and control is vaccine immunization. Vaccination can effectively reduce the spread of the virus and avoid greater economic losses. With the rapid development of molecular biology, viral gene deletion mutants are promising vaccine candidates for the control and eradication of infection. At present, the gE gene is the preferred target gene for the construction of attenuated vaccines, and the construction of gE gene deletion vaccines based on mutant viruses has always been a research hotspot.

The constructed gE deletion mutant has good safety and immunogenicity, and significantly reduces the replication and proliferation of the virus in the central nervous system. Following intranasal (i.n.) or intramuscular (i.m.) vaccination of foals with gE-deficient EHV-1, there were no clinical symptoms following vaccination and all serum neutralizing antibody titers were significantly increased (Tsujimura et al., 2009). The gE-deleted marker vaccine strain BoHV-1ΔgEβgal was generated by homologous recombination, replacing the viral gE gene with β-galactosidase (βgal). After inoculation with BoHV-1ΔgEβgal, the animal body can induce specific humoral and cellular immune responses, which can resist the re-attack of the virus, and the shed virus cannot be detected in the nasal secretions (Romera et al., 2014; Petrini et al., 2020). After vaccination with PRV gE-deficient vaccine, the body can produce high levels of neutralizing antibodies, providing clinical protection and significantly reducing viral shedding after infection (Wang et al., 2014, 2015a, 2016). The intracellular carboxy-terminus of VZV gE affects the transport of gE, which in turn affects the final presentation pathway of gE as an antigen. Following mutations at the carboxy-terminus of gE (mutant Y569A and mutants S593A, S595A, T596A and T598A), the ability to induce the highest gE-specific IgG titers reduced viral transmission(Cao et al., 2021). Furthermore, gE deletion mutants are defective in transmission from epithelial cells to axons and from neuronal cell bodies to axon terminals. After infection of epithelial cells, the virus spreads to neurites and propagates to neuronal cell bodies by retrograde axonal transport. Compared with HSV-1, retrograde spread of virions was reduced 100-fold when gE was deleted (McGraw and Friedman, 2009). When HSV-2 gE-deleted mutants were inoculated into animal brains, they were 5 orders of magnitude less virulent than wild-type viruses (Awasthi et al., 2012).

The gE/gI double deletion, thymidine kinase (TK) and gE double deletion or TK, gE and gI triple deletion strains may serve as promising vaccine candidates against emerging variants. The TK gene is a virulence-related gene and is used to treat viral infections (Xie et al., 2019). The candidate vaccine of the gE/gI double deletion strain was not pathogenic to suckling piglets and produced significantly higher levels of neutralizing antibodies than the PRV Bartha-K61 vaccine (Gu et al., 2015). Likewise, inoculation of piglets with EGFP-labeled gE/gI-free PRV was able to induce high levels of gB-specific antibodies, and the vaccinated piglets did not have any clinical symptoms and only developed mild fever 7 days after lethal challenge (Yin et al., 2017). Piglets provided complete protection against challenge only 7 days after vaccination with different doses of TK and gE double deletion PRV mutants, superior to PRV Bartha-K61 vaccine (Wang et al., 2018b). Compared with the Bartha-K61 vaccine group, piglets vaccinated with PRV gE, gI and TK triple deletion mutants could induce high levels of neutralizing antibodies, which were able to resist the lethal PRV challenge (Hu et al., 2015; Zhang et al., 2015).

The triple deletion mutants of gE, gl and TK had better safety and immunogenicity than the double deletion mutants of gE and gI, further demonstrating that deletion of the TK gene reduced virus virulence. After piglets were inoculated with gE, gI and TK triple deletion mutant rZJ01ΔTK/gE/gI and gE and gI double deletion mutant rZJ01ΔgE/gI, the two groups produced similar levels of neutralizing antibodies, both of which were resistant to ZJ01 challenge, the detected viral level in the brains of piglets in the rZJ01ΔTK/gE/gI group was significantly lower than that in the rZJ01ΔgE/gI group (Dong et al., 2017). This conclusion was also confirmed in mice and sheep (Cong et al., 2016).

The deletion mutant virus has a non-essential region for replication, which can be used as an insertion site for exogenous genes, and has the conditions to be used as a concatenated recombinant live vaccine vector. Safe and immunogenic gE/gI/TK-deleted PRV mutants can be used as vaccine vectors to construct recombinant viruses expressing classical swine fever virus (CSFV) E2 protein. The recombinant virus rPRVTJ-delgE/gI/TK-E2 expressing classical swine fever virus (CSFV) E2 protein was generated based on the triple deletion mutant rPRVTJ-delgE/gI/TK of gE, gI and TK. After immunization with recombinant virus rPRVTJ-delgE/gI/TK-E2, pigs showed no clinical symptoms and no virus shedding, and the anti-PRV neutralizing antibodies and anti-CSFV neutralizing antibodies produced by pigs could resist the mutant PRV TJ strain and CSFV Shimen strain attack (Lei et al., 2016).

In addition, gE-deletion-marked vaccines can decontaminate and eradicate epidemics because it can distinguish vaccinated animals from naturally infected animals. The gE-deleted vaccine can be distinguished from wild virus infection or vaccine immunity by the antibodies produced after vaccination. The BHV-1 gE-deleted vaccine is the most widely used labeled vaccine, using serum collected at approximately 6-month intervals for 5 consecutive years, serologically screened for gE-specific antibodies by ELISA to eradicate BHV-1 infection (Tignon et al., 2017; Alkan et al., 2018; Petrini et al., 2019). The gE CTD protein can be used as an indicator antibody and coating antigen for the gE CTD-specific blocking ELISA test to distinguish between BHV-1 wild strains or gE CTD deletion strain-infected calves (Chowdhury, 2016). Piglets inoculated with JS-2012-ΔgE/gI also failed to produce PRV-specific gE-ELISA antibodies (Tong et al., 2016). Dual real-time recombinase polymerase amplification (RPA) assays rapidly identify wild-type PRV and gE-deleted vaccine strains. Specific primers and probes are designed for the conserved regions of the genome (such as gB, gE, gD genes), which can specifically detect and identify wild-type strains and gE-deficient vaccine strains (Wernike et al., 2011; Wang et al., 2018a). An EvaGreen-based modified multiplex real-time polymerase chain reaction (EGRT-PCR) assay that successfully differentiates wild-type and gE-deficient BoHV-1 strains based on gene-specific melting temperature (Tm) peaks (Pawar et al., 2017). In addition, the loop-mediated isothermal amplification assay can also rapidly identify wild-type and gE-deficient strains (Nemoto et al., 2010; Zhang et al., 2010a; Pawar et al., 2015).

Based on its attenuation, immunogenicity, and protection after challenge, gE-deletion-labeled vaccines are effective and safe whether used as live attenuated or inactivated vaccines.

## Conclusion and direction

gE is an important virulence gene that affects virus virulence in different ways. In this review, we summarize reports on gE structure and gE/gI heterodimers, gE interactions with other proteins of the virus, and passive immunization of emerging viruses with gE-depleted vaccines. However, the molecular mechanisms of how gE interacts with envelope proteins or other proteins, and how they affect the secondary envelope of the virus, cell-to-cell transmission and other related biological properties remain to be further studied. These will help explain the pathology of the virus and develop drugs that prevent the virus from spreading.

In addition, the gE ETD of VZV can act as a specialized viral receptor that binds to insulin-degrading enzyme (IDE). IDE is a zinc metalloprotease that is present in the cytosol of all cells, degrades many small proteins, including insulin and amyloid, and is also involved in the pathogenesis of diabetes and Alzheimer’s disease (AD). Binding of aglycosylated precursors of VZV gE proteins in IDE and ER is important for cell-to-cell transmission and cell-free viral infection (Li et al., 2006, 2010; Ali et al., 2009; Carpenter et al., 2010; Bernstein et al., 2020). Maybe this theory can be used for further research on AD. The research on VZV gE-IDE in AD is still in its infancy, and there are still many problems that remain unsolved and need further research. Unlike most *alphaherpesviruses*, the *gE* gene of MDV is an essential gene for infecting cells and replicating *in vitro*, and deletion results in the inability of virions to spread from infected cells to uninfected cells (Schumacher et al., 2001). It is involved in the spread of the virus between cells and is the main antigen recognized by the host immune system. It can induce the body to produce neutralizing antibodies against MDV infection, mediate the host’s immune evasion and enhance the virulence of MDV. A recombinant cell line was established with gE to produce CVI988 vaccine, which can resist the challenge of highly virulent MDV-I EU-I after immunizing chickens (Schumacher et al., 2002). Studies have shown that the *gE* gene of MDV I attenuated strain 814 has different degrees of mutation, and these sites can be used for the design of future MDV attenuated vaccines (Zhang et al., 2012).

Many herpes viruses continually mutate and change their virulence, and traditional vaccination provides only partial protection, leading to small outbreaks of the disease (Tong et al., 2015; Wang et al., 2015b; Sun et al., 2018). In order to prevent and control the spread of diseases, and with the rapid development of molecular biology, in-depth research on virus characteristics, virus latent infection and immune mechanisms, a variety of new vaccines, including gene deletion vaccines, are being further developed. Gene deletion vaccines use genetic engineering techniques to remove specific genes associated with viral virulence, but maintain the immunogenicity of the virus. The deletion site of the gene deletion vaccine is clear, and it has the advantages of stable virulence, less detoxification, and significantly reduced replication in most of the central nervous system. The *alphaherpesvirus* genome is large and contains many coding genes. Among them, TK, gC, gE, gI, have all been proved to be non-essential genes for virus replication. gE is a common missing target gene that has been used in vaccine candidates over the past few decades and remains available as long as new immunization strategies are developed. gE has also been used in the study of recombinant vaccines, and since gE is an essential neurovirulence factor in animal models, its specific antibodies are readily detected in organisms with varicella, and T cell-mediated immunity against gE has been demonstrated (CMI) epitope, based on which the adjuvanted recombinant glycoprotein E herpes zoster vaccine developed is superior to the traditional HZ live attenuated vaccine (Levin and Weinberg, 2020). However, there are also certain shortcomings, so it is of great significance to develop new and efficient vaccines. In addition, we need further research on vaccines to balance cell-mediated immune responses and humoral immune responses to improve the protection rate and antigen expression levels in immunized animals.

## Author contributions

YN and YH contributed ideas for the review, wrote the manuscript, and produced the figures. AC, MW, QY, YW, BT, XO, JH, SM, DS, XZ, SZ, QG, SC, ML, DZ, and RJ edited and revised the manuscript. All authors contributed to the article and approved the submitted version.

## Funding

This work was supported by the National Natural Science Foundation of China (32072894), the China Agriculture Research System of MOF and MARA, and the Sichuan Veterinary Medicine and Drug Innovation Group of China Agricultural Research System (SCCXTD-2020-18).

## Conflict of interest

The authors declare that the research was conducted without any commercial or financial relationships that could be construed as a potential conflict of interest.

## Publisher’s note

All claims expressed in this article are solely those of the authors and do not necessarily represent those of their affiliated organizations, or those of the publisher, the editors and the reviewers. Any product that may be evaluated in this article, or claim that may be made by its manufacturer, is not guaranteed or endorsed by the publisher.
